# Stress-Induced Enhancement of Mouse Amygdalar Synaptic Plasticity Depends on Glucocorticoid and ß-Adrenergic Activity

**DOI:** 10.1371/journal.pone.0042143

**Published:** 2012-08-10

**Authors:** Ratna Angela Sarabdjitsingh, Daniel Kofink, Henk Karst, E. Ron de Kloet, Marian Joëls

**Affiliations:** 1 Department of Neuroscience and Pharmacology, Rudolf Magnus Institute of Neuroscience, University Medical Center Utrecht, Utrecht, The Netherlands; 2 Division of Medical Pharmacology, Leiden/Amsterdam Centre for Drug Research/Leiden University Medical Centre, University of Leiden, Leiden, The Netherlands; Neuroscience Campus Amsterdam, VU University, The Netherlands

## Abstract

**Background:**

Glucocorticoid hormones, in interaction with noradrenaline, enable the consolidation of emotionally arousing and stressful experiences in rodents and humans. Such interaction is thought to occur at least partly in the basolateral nucleus of the amygdala (BLA) which is crucially involved in emotional memory formation. Extensive evidence points to long-term synaptic potentiation (LTP) as a mechanism contributing to memory formation. Here we determined in adolescent C57/Bl6 mice the effects of stress on LTP in the LA-BLA pathway and the specific roles of corticosteroid and β-adrenergic receptor activation in this process.

**Principal Findings:**

Exposure to 20 min of restraint stress (compared to control treatment) prior to slice preparation enhanced subsequent LTP induction in vitro, without affecting baseline fEPSP responses. The role of glucocorticoid receptors, mineralocorticoid receptors and β2-adrenoceptors in the effects of stress was studied by treating mice with the antagonists mifepristone, spironolactone or propranolol respectively (or the corresponding vehicles) prior to stress or control treatment. In undisturbed controls, mifepristone and propranolol administration in vivo did not influence LTP induced in vitro. By contrast, spironolactone caused a gradually attenuating form of LTP, both in unstressed and stressed mice. Mifepristone treatment prior to stress strongly reduced the ability to induce LTP in vitro. Propranolol normalized the stress-induced enhancement of LTP to control levels during the first 10 min after high frequency stimulation, after which synaptic responses further declined.

**Conclusions:**

Acute stress changes BLA electrical properties such that subsequent LTP induction is facilitated. Both β-adrenergic and glucocorticoid receptors are involved in the development of these changes. Mineralocorticoid receptors are important for the maintenance of LTP in the BLA, irrespective of stress-induced changes in the circuit. The prolonged changes in BLA network function after stress may contribute to effective memory formation of emotional and stressful events.

## Introduction

Emotionally arousing and stressful experiences are generally well remembered [1]. Such effective memory for stress-related information is considered to be adaptive [2], [3]. The primary bodily response during stressful experiences involves the activation of the autonomous nervous system which (indirectly) increases levels of noradrenaline, acting via β-adrenoceptors in multiple memory-related brain areas such as the amygdala, hippocampus and prefrontal cortex [4]. Slightly later the hypothalamic-pituitary-adrenal (HPA) axis is activated, which triggers the release of glucocorticoid hormones (cortisol in humans, corticosterone in rodents) from the adrenal cortex [5]. Glucocorticoid hormones readily enter the brain and exert rapid nongenomic and slow genomic actions via membrane-bound and nuclear variants respectively of the mineralocorticoid and glucocorticoid receptor (MR and GR) [6], [7], [8]. Both receptor types are abundantly expressed in structures essential for learning, memory formation and emotional behaviour. The MR has a 10-fold higher affinity for corticosterone than the GR, rendering brain areas expressing MR and GR responsive to both basal and stress-induced levels of corticosterone [9], [10].

Studies in humans and rodents suggest that stress effects on emotional processing and memory formation are largely mediated by the amygdala [1], [3]. Specifically, the basolateral nucleus (BLA) has been suggested as a locus for memory storage of stressful experiences [11], [12], [13]. Glucocorticoids enhance the consolidation of emotionally arousing experiences and this requires arousal-induced noradrenergic activation of BLA circuits [13], [14], [15], [16], presumably via a cAMP-dependent protein kinase pathway [17]. The BLA also acts as a critical gateway in mediating stress effects on other aspects of memory formation, via projections to structures such as the hippocampus and prefrontal cortex [2], [11], [14], [18], [19]. In the human brain, glucocorticoid hormones are known to change amygdalar vigilance, and -depending on the delay between steroid exposure and task performance- alter reactivity and coupling with some of these structures [20].

The changes in BLA cell and circuit function underlying stress-induced facilitation of emotional memory formation and the role of noradrenaline and glucocorticoids in this process are still largely unknown. Such changes probably target long-term strengthening of synaptic contacts (long-term potentiation, LTP), which is thought to be critical in learning and memory formation [21], [22]. Although the effects of stress and glucocorticoids on hippocampal LTP have been extensively documented (reviewed by [19]), few studies have addressed this question in the BLA and the results so far have been equivocal, sometimes even within the same lab. Two studies described that stress facilitates LTP in the BLA [23], [24], while other studies reported a reduction or no effect on LTP [25], [26], [27].

Here we examined if a brief period of restraint stress changes i) field responses evoked 1–4 hrs later in vitro in the BLA by stimulation of the lateral amygdala and ii) the ability to induce LTP in this pathway. We subsequently used a pharmacological approach to determine the contribution of MR, GR and β-adrenergic receptor activation in these changes.

## Materials and Methods

### Animals

Male C57/Bl6 mice (Harlan, the Netherlands; 5–6 weeks old at the moment of arrival) were group-housed in a temperature- and humidity-controlled room with water available *ad libitum*. After their arrival, animals were left undisturbed, to acclimatize for approximately 1 week. The studies were performed early in the morning when endogenously circulating corticosterone levels were still low.

### Ethics Statement

All experiments were approved and conducted according to the guidelines of the Animal Committee for Bioethics of the University of Utrecht (Permit number 2010.I.11.236). All efforts were made to minimize suffering.

### Experimental design

In the first series of experiments, the effect of acute stress on BLA synaptic plasticity was studied in mice that were restrained for 20 min (n = 6–9). Restraint stress was applied by placing mice individually in a transparent plexiglas cylinder (5 cm diameter) provided with ample air holes for ventilation. Subsequently, a small round-shaped lid was used to fixate the mouse inside the tube, thereby preventing movement but allowing the animal to breathe freely; control mice were left undisturbed. Directly after the stressor, the mice were taken out of the restrainer and rapidly decapitated after which brains and trunk blood was collected. Compared to undisturbed controls, mice exposed to restraint stress showed significantly elevated corticosterone plasma levels (controls, 9.2±1.3 ng/ml vs stress, 399.0±23.6 ng/ml, p<0.0001, as determined in trunk blood, using a commercially available radioimmuno assay (MP Biomedicals Inc., CA., USA)); this confirms that this stressor is indeed an potent activator of HPA axis responses.

**Figure 1:**
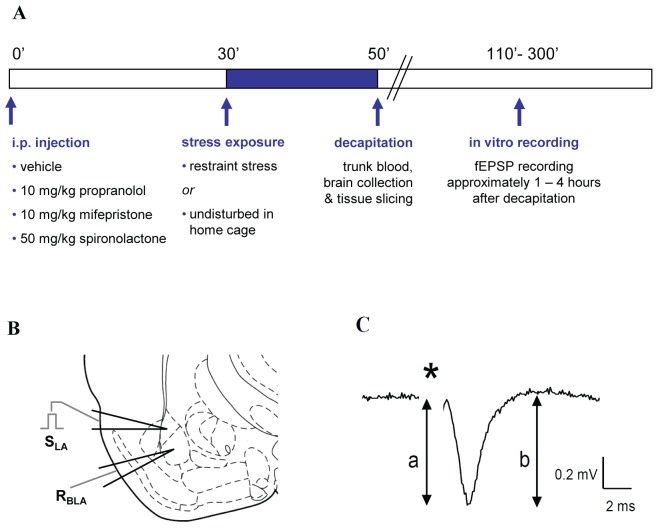
Schematic overview of the experimental design and method. **A** Schematic time line of the experimental design. Thirty minutes after injection with vehicle or antagonist, mice were subjected to either restraint stress or left undisturbed. After another 20 min, animals were rapidly decapitated and brains were collected for slice preparation. Approximately 1–4 hours after decapitation, in vitro electrophysiological recordings were carried out. **B** Positioning of the stimulation (S_LA_) and the recording electrode (R_BLA_) at their sites within the lateral (LA) and the BLA respectively in mouse coronal brain slices. **C**) Representative local fEPSP evoked by stimulation of the LA–BLA pathway. The amplitude of the signal was calculated according to the formula (a+b)/2 as indicated in the figure. * indicates position of the stimulus artefact.

In the second series, the contribution of i) corticosterone acting via MR or GR and ii) β-adrenergic actions via the β2-adrenoceptor was investigated. To selectively block these receptors, animals were pretreated intraperitoneally with the specific antagonists spironolactone (50 mg/kg in propylene glycol Sigma-Aldrich, Germany), mifepristone (10 mg/kg in propylene glycol, Sigma-Aldrich, Germany) and propranolol (10 mg/kg in 0.9% NaCl, Sigma-Aldrich, Germany) respectively, or vehicle (propylene glycol, Sigma-Aldrich, Germany or 0.9% NaCl). Thirty minutes later and similar as described above, animals were exposed to either 20 min of restraint stress or left undisturbed and subsequently rapidly decapitated to collect the brains and trunk blood. A schematic overview of the experimental design is depicted in Fig. 1A. Drug doses were chosen based on literature and earlier shown to effectively block MR, GR or β2-adrenoceptor mediated effects [28], [29], [30].

### Electrophysiology

After rapid dissection, the brain was chilled in ice-cold artificial cerebrospinal fluid (aCSF) consisting of 120 mM NaCl, 3.5 mM KCl, 5.0 mM MgSO_4_, 1.25 mM NaHPO_4_, 0.2 mM CaCl_2_, 10 mM D-glucose, and 25 mM NaHCO_3_, gassed with 95% O_2_ and 5% CO_2_. Coronal brain slices (350 µm thick) containing both the lateral amygdala and the BLA were prepared using a Leica VT1000S Vibratome. All slices were collected and submerged in aCSF in a holding chamber for 1–4 hours before being transferred to the recording chamber, maintained at 32°C (Fig. 1A). BLA field excitatory postsynaptic potentials (fEPSP) were evoked by stimuli delivered to the afferent fibres via a bipolar tungsten electrode insulated to the tip (0.075 mm µm tip diameter) and positioned in the lateral amygdala which supplies one of the major afferent pathways to the BLA (Fig. 1B) [31]. For recording, glass microelectrodes filled with aCSF (2–3 MΩ) were used. Single pulses (0.15 ms) were delivered at a rate of once per 30 s (Neurolog digital stimulator, Cambridge Electronic Design, United Kingdom) and amplified with a gain of 1,000. The stimulation intensity was adjusted to produce a fEPSP of approximately 50% of the maximal amplitude. Tetanic stimulation was applied only when responses to single stimuli had remained stable for at least 20 min. Subsequently, as previously described [32], stable non-saturated LTP was induced by applying one train of high-frequency stimulation (100 Hz, 1 s). Synaptic responses were further monitored for 60 min post-tetanus. The amplitude of the fEPSP was calculated as (a+b)/2 with (a) being the difference between the sharp negative voltage deflection at the onset and the negative peak, and (b) the difference between the negative peak and the succeeding positive peak (Fig. 1C) [33], [34]. Two consecutive traces were averaged to represent the mean per minute. Data were acquired, stored, and analysed using Signal 2.16 (Cambridge Electronic Design, United Kingdom). Changes in synaptic strength were expressed relative to normalized baseline (average of 20 min pre-tetanus) and expressed as mean ± SEM.

### Data analysis

All statistical analyses were carried out with SPSS version 16.0 (SPSS, Gorinchem, The Netherlands). A two-tailed paired Student's t-test was used to compare synaptic responses before versus tetanization within each group. For between-group comparisons assessing the drug-induced effects on LTP, data were analysed using one-way ANOVA or the general linear model for repeated measures (GLM) where appropriate. Where applicable, pairwise post-hoc comparisons were carried out using a Tukey's or Bonferroni's post-hoc test. To assess the effect of stress or drug treatment on the means of baseline transmission and LTP, the significance of the difference between the means was calculated by two-tailed unpaired Student's t-test (experiment 1) or one-way ANOVA (experiment 2). Probability values of p<0.05 were considered to represent significant differences.

## Results

### Effect of stress on baseline synaptic transmission

**Figure 2:**
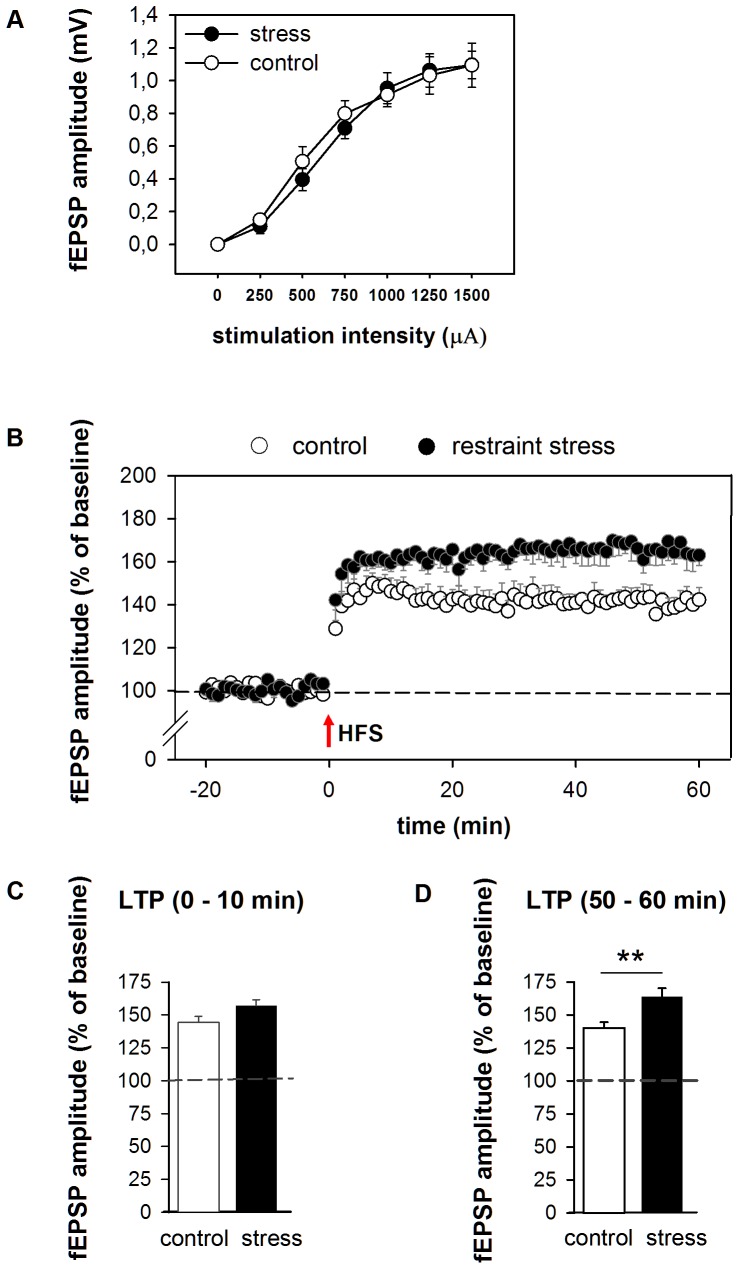
The effect of restraint stress on baseline transmission and LTP. **A** Input-output curves constructed from the fEPSP amplitude vs increasing stimulation intensities at the BLA from brain slices of control (open symbols, n = 8) and restrained stressed mice (filled symbols, n = 7). No significant differences in stimulus-response relationships or maximal fEPSP amplitude were found. **B** HFS (1×100 Hz, 1 s) of the LA afferents resulted in stable LTP at BLA synapses in slices of control mice which was more pronounced in stressed mice. Averaged mean values during **C** 0–10 min and **D** 50–60 min of the post-tetanus recording period indicate that compared to controls (white columns), late LTP is significantly enhanced in stressed mice (black columns). Dashed line indicate pre-tetanus baseline levels. Error bars indicate SEM. ** p<0.01.

We first examined whether basal synaptic field responses in the BLA were altered by stress exposure, by comparing stimulus-response relationships for evoked fEPSP amplitudes obtained in brain slices from control and stressed mice. No significant differences in the overall input-output curves were found (p = 0.72) between brain slices from control versus stressed mice (Fig. 2A). The averages of the half maximal fEPSP amplitude evoked during baseline recordings (p = 0.66) or the stimulation intensity to produce this fEPSP (p = 0.52) also did not reveal any significant differences (Table 1), suggesting that acute stress does not affect baseline transmission.

**Table 1:** The baseline amplitudes and stimulation intensities of the half maximal fEPSPs in the BLA in the different experimental groups.

Group	N	Baseline amplitude (mV)	Stimulation intensity (µA)
Control	8	0.66±0.05	575±55
Stress	7	0.63±0.03	532±36
0.9% NaCl (vehicle)	7	0.64±0.08	471±52
0.9% NaCl+stress (vehicle)	7	0.56±0.02	498±56
Propylene glycol (vehicle)	8	0.60±0.05	498±52
Propylene glycol+stress (vehicle)	7	0.65±0.04	463±35
Vehicle (pooled)	15	0.62±0.04	484±46
Propranolol	6	0.61±0.01	534±43
Mifepristone	6	0.65±0.08	486±37
Spironolactone	7	0.63±0.06	398±32
Vehicle+stress (pooled)	14	0.61±0.03	484±36
Propranolol+stress	6	0.62±0.05	452±37
Mifepristone+stress	8	0.59±0.02	460±36
Spironolactone+stress	8	0.64±0.05	485±37

Note. Table 1 shows that neither stress nor pharmacological manipulations prior to stress affect baseline transmission. Relevant comparisons for each experiment are described in the text.

### Effect of stress on LTP in the amygdala

We next investigated whether restraint stress would affect synaptic plasticity induced by high frequency stimulation, i.e. one 1 s train of 100 Hz stimuli. This stimulation paradigm is known to effectively induce a stable, non-saturated form of LTP in the BLA [32], which was confirmed in the present study (Fig. 2B). In brain slices of both control and stressed mice, high frequency stimulation (HFS) evoked substantial potentiation of the half-maximal amplitude compared to the pre-tetanus baseline (mean fEPSP amplitude ± SEM over the entire 60 min post tetanus period vs baseline; p<0.001 in both cases). The degree of synaptic potentiation was more pronounced in slices from mice exposed to restraint stress compared to control mice (mean fEPSP amplitude ± SEM over the entire 60 min post tetanus period: control 142.1±3.7% vs stress 163.2±4.0%; p<0.001).

This was mostly carried by a significant difference between the two groups at a later stage of LTP (t = 50–60 min, Fig. 2D, p<0.01); immediately after HFS (t = 0–10 min, Fig. 2C), there was only a trend towards significance between stressed and control mice in the average level of post-tetanic potentiation (control 144.3±4.6% vs stress 157.0±4.6%; p = 0.07). Collectively, these results suggest that acute stress changes the network function in the BLA such that >1 hr later induction of long-term (but not short-term) synaptic plasticity is altered in the BLA.

### Effect of vehicle treatment

To test the hypothesis that the enhancing effects of stress on BLA LTP result from corticosterone and/or noradrenalin acting on the corticosteroid receptors MR and GR or the β2-adrenoceptor, respectively, animals had to be pretreated with specific receptor antagonists. We first assessed whether the intraperitoneal injection itself could potentially affect baseline transmission or LTP formation. To assess this, we studied vehicle (0.9% NaCl or propylene glycol) pre-treated animals that were either left undisturbed or subjected to stress. Compared to the non-injected controls, baseline fEPSP amplitude or stimulation intensity did not differ in vehicle pre-treated non-stressed animals (Table 1; *F*_(2,20)_ = 0.3, p = 0.76 and *F*_(2,20)_ = 1.31, p = 0.29;) or in vehicle pre-treated stressed animals (*F*_(2,24)_ = 0.62, p = 0.54 and *F*_(2,24)_ = 2.6, p = 0.1), respectively. The values for the magnitude of average LTP formation were also highly comparable for the two vehicles under non-stress (mean post-tetanic fEPSP amplitude ± SEM: control 142.1±3.7% vs. 0.9% NaCl 140.0±5.4% vs propylene glycol 137.8±2.5%; *F*_(2,20)_ = 0.30, p = 0.74) or stress conditions (mean post-tetanic fEPSP amplitude ± SEM: stress 163.2±4.0% vs. 0.9% NaCl 159.8±2.3% vs propylene glycol 157.5±2.2%; *F*_(2,19)_ = 0.70, p = 0.51). Collectively, these data suggest that 1) neither baseline transmission nor synaptic plasticity was affected by injection of saline or propylene glycol compared to non-injected mice, and 2) that the degree of potentiation was highly comparable for the two vehicle conditions. In the remainder of the analyses, the two vehicle groups were therefore combined and compared with spironolactone-, mifepristone- and propranolol-treated mice.

### Effect of receptor blockade in non-stressed mice

We first investigated whether drug treatment affects baseline transmission of BLA neurons in non-stressed animals. One-way ANOVA analysis showed no significant differences between the experimental groups with respect to the half maximal stimulation intensities (*F*_(3,32_) = 0.79, p = 0.51) and fEPSP amplitude (*F*_(3,29)_ = 0.07, p = 0.98; Table 1), indicating that the in vivo antagonist pretreatment did not change baseline transmission.

**Figure 3:**
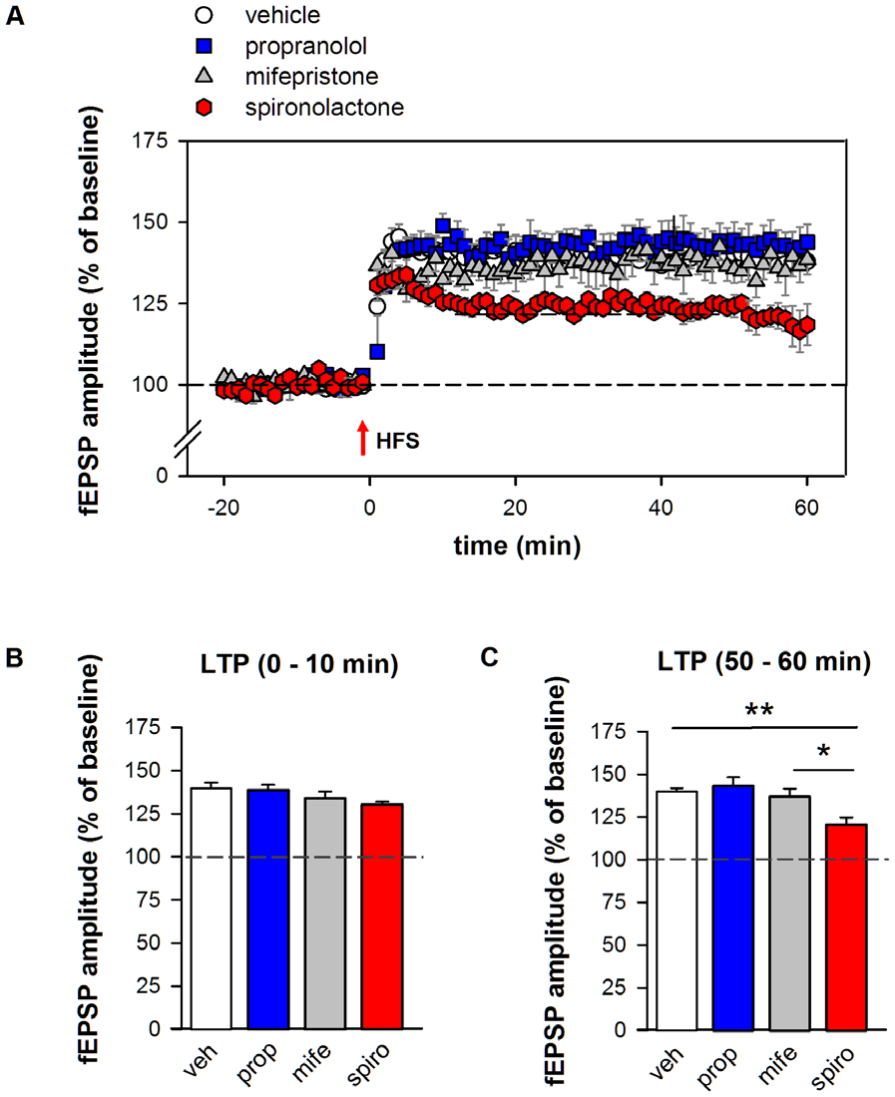
BLA LTP and antagonist pretreatment in undisturbed mice. **A** HFS evoked potent and stable LTP at BLA synapses in all experimental groups. Compared to vehicle injected mice (n = 15, white circles), BLA LTP was not affected by propranolol (n = 6, blue circles) or mifepristone pretreatment (n = 6; grey circles). Pretreatment with spironolactone (n = 7, red circles) however gradually attenuated LTP induced some hours later in vitro. **C** Bar chart illustrating the averages per treatment group for 0–10 min and **D** 50–60 min of the post-tetanus period, showing attenuated LTP in spironolactone treated mice at the later time-point. Dashed line indicates pre-tetanus baseline values. Error bars indicate SEM. Tukey's post hoc test * p<0.05, ** p<0.01.

Subsequently, we studied whether synaptic plasticity was affected in these animals. As indicated in Fig. 3A, compared to their individual pre-tetanus baseline values HFS resulted in increased synaptic responses in all groups which sustained over the entire 60 min post tetanus period (p<0.001). To investigate the pattern and level of potentiation in more detail, mixed ANOVA including repeated measures at the different time points after HFS was used. This revealed a significant effect of drug pretreatment on the amount of LTP that was induced due to HFS (*F*_(3,30)_ = 6.38, p<0.01). Tukey's post hoc analysis indicated that spironolactone pretreatment amounted to significantly less LTP (mean fEPSP amplitude ± SEM over the 60 min posttetanus period: 124.0±3.2%), compared to vehicle (139.5±3.1%, p<0.01). No significant effect of post-tetanus time (*F*_(6,23; 187,6)_ = 1.55, p = 0.16) or a treatment×time interaction (*F*_(18,76; 187,6)_ = 1.44, p = 0.11) on BLA synaptic plasticity was found, indicating no difference between the groups in this respect.

Further detailed analysis of the LTP curve showed that the initial responses of all groups after HFS (t = 0–10 min) were comparable to the vehicle control group (Fig. 3B, *F*_(3,30)_ = 1.4, p = 0.26), but that LTP gradually attenuated in the spironolactone group, resulting in a significantly reduced potentiation in the time-interval t = 50–60 min (Fig. 3C, *F*_(3,28)_ = 7.70, One-way ANOVA p<0.001, control: 139.0±1.5% vs spironolactone: 120.3±4.4%, Tukey's post hoc test p<0.01). Additionally, a significant reduction in BLA LTP was present when compared to mifepristone (spironolactone: 120.3±4.4%, 136.7±4.8%, Tukey's post hoc test p<0.05), suggesting unstable LTP formation specifically when MR, and not GR, is blocked. Synaptic plasticity was not affected by propranolol or mifepristone treatment.

### Effect of receptor blockade in stressed mice

To study the role of MR, GR and the β-adrenoceptor in the stress-induced enhancement in BLA LTP, animals were pretreated with specific antagonists prior to stress exposure. This pharmacological pretreatment did not affect fEPSP amplitude (*F*_(3,36_) = 0.28, p = 0.84) or half-maximal stimulation intensity (*F*_(3,40)_ = 1.37, p = 0.27; Table 1), suggesting similar properties in BLA baseline transmission across the experimental groups.

**Figure 4:**
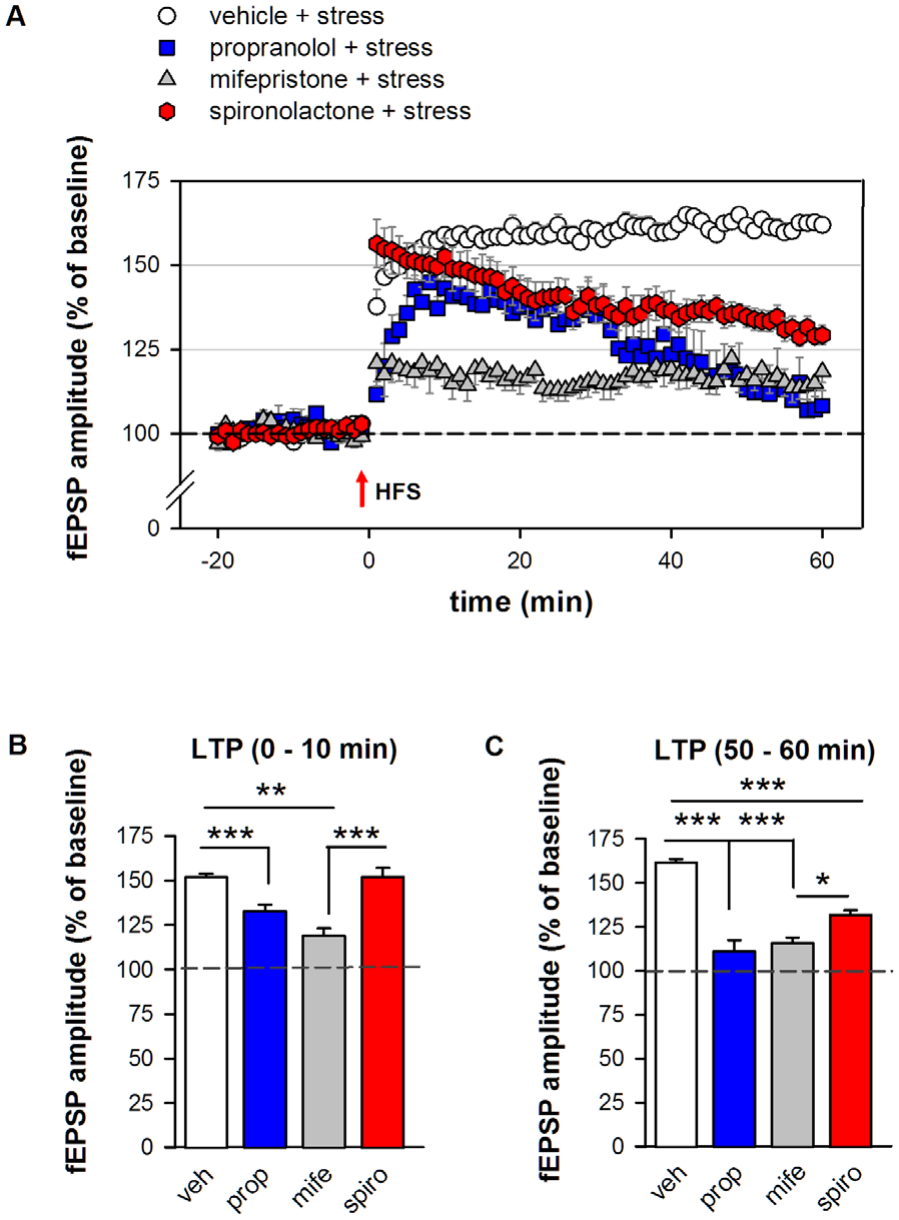
BLA LTP in acutely stressed mice pretreated with antagonists. **A** HFS resulted in attenuated and unstable LTP in mice injected with propranolol (n = 6; blue circles) or spironolactone (n = 8; red circles) before stress exposure. Mifepristone pretreatment (n = 8; grey circles) attenuated LTP compared to vehicle (n = 14, white circles). **C** Bar chart illustrating the averages per treatment group for 0–10 min and **D** 50–60 min of the post-tetanus period. Dashed line indicates pre-tetanus baseline values. Error bars indicate SEM. Tukey's post hoc test * p<0.05, ** p<0.01, *** p<0.001.

Compared to their pre-tetanus baseline values, high frequency stimulation resulted in significant LTP formation in all groups, averaged over the entire post-tetanus period (p<0.001). Mixed ANOVA with repeated measures on the time points after HFS showed multiple results, including time (*F*_(59, 1888)_ = 6.55, p<0.001), antagonist pretreatment (*F*_(3, 32)_ = 43.60, p<0.001) and an interaction effect (*F*_(177, 1888)_ = 5.19; p<0.001). Stressed animals pretreated with spironolactone (Fig. 4A) exhibited a qualitatively comparable LTP pattern as observed in non-stressed spironolactone pretreated mice (Fig. 3A), showing initially a clear post-tetanic potentiation, which developed into attenuated long-term potentiation. The attenuation was statistically significant in spironolactone animals compared to the vehicle-treated controls (Fig. 4C, for all groups: t = 50–60 min *F*_(3,32)_ = 61.72, p<0.0001; Tukey's post hoc test: vehicle 161.5±1.9% vs spironolactone 131.7±2.9%, p<0.001), and mifepristone treated animals (Tukey's post hoc test: spironolactone 131.7±2.9% vs mifepristone 115.9±3.0%, p<0.05). Pretreatment with mifepristone largely impaired the development of LTP, suggesting that GR blockade not only prevented the stress-induced enhancement (Fig. 4A, overall post tetanic fEPSP mean *F*_(3,32)_ = 43.60, p<0.001; Tukey's post hoc test: vehicle: 159.1±1.5% vs mifepristone: 116.8±2.7%; p<0.001), but also reduced LTP beyond non-stressed control levels (vehicle: 139.5±3.1% vs mifepristone: 116.8±2.7%; p<0.001). Propranolol decreased the initial (t = 0–10 min) stress-induced enhancement (Fig. 4B, *F*_(3,31)_ = 21.28, p<0.001, vehicle vs propranolol, p<0.01), but also affected the later phase of LTP (t = 50–60 min) which was not different from pre-tetanus baseline values (p = 0.14), resulting in an overall impaired synaptic strengthening (Fig. 4C, *F*_(3,32)_ = 61.72, p<0.001, vehicle vs propranolol p<0.001).

Altogether, the data suggests that pharmacologic blockade of MR, GR or the β-adrenoceptor prior to stress affects BLA synaptic plasticity 1–4 hours later by impairing induction and stability of LTP.

## Discussion

Emotional aspects of stressful events are very well retained [1], [35], [36]. Behavioural studies have shown that this phenomenon critically depends on the BLA and involves noradrenergic and corticosteroid-mediated signalling [3], [13], [14], [15], [37], [38]. Synaptic plasticity is thought to be the principal mechanism by which such memory formation is accomplished, not only in hippocampal regions [19], [21], [22], but also in the amygdala [39], [40], [41]. In the current study we probed the mechanisms through which stress promotes the retention of emotional information in the BLA, by testing baseline activity and the ability to induce LTP in the period during which this information is supposedly consolidated; moreover, we examined the specific role of the β-adrenoceptor, MR and GR in these processes.

The main conclusions are that 1) stress did not change basal transmission in the BLA as measured 1–4 hours later at the fEPSP level in response to LA stimulation, at least not at the level of field potential recording. However, 2) network function in the BLA was still changed, because LTP was more effectively induced after stress. 3) Blocking β-adrenoceptors or GR at the time of stress exposure did not change basal transmission (at least not when examining fEPSP amplitudes) but 4) did reduce the development of LTP, even beyond the levels seen under control conditions. 5) MR seems to be necessary for the development of LTP in the BLA, regardless of earlier stress exposure.

Despite the well-documented behavioural effects, very few studies so far have addressed the BLA circuit function after stress. One series of studies, mainly from the same lab, examined BLA basal fEPSP amplitudes and synaptic plasticity in vivo (evoked by stimulation of various input pathways) in anaesthetized rats earlier exposed to predator stress [42], [43], an elevated plus maze [24], [26], corticosterone administration [26] or (repeated) swim stress [27]. Basal transmission was enhanced in only one of these studies [27], while the other studies did not report any changes in basal transmission. LTP was found to be enhanced several hours post-stress in some studies [24], [42], [43], but reduced in another [26]. Why these studies have such diverging results remains speculative. Most likely this discrepancy can be attributed to the different use of species, context (in vivo vs in vitro recordings), input pathways and various types of stressors among the various studies. One important factor may be the duration and severity of stress used. Repetitive exposure to stress was found to decrease amygdalar synaptic plasticity [27], while exposure to a single, acute stressor appears to increase BLA LTP, as presently shown by us and previously by others [43].

In one study rats were exposed to a single 30 min period of restraint stress , comparable to our design, but examined in vitro 24 hrs after the stressor, i.e. at a much later point in time than tested by us. Stimulation of the external capsula caused multispike responses in the BLA, pointing to a reduced GABAergic tone, and a facilitated induction of LTP. Together with the current findings, this may indicate that stress could change local network function over the course of hours so that LTP induction is enhanced, an effect that might last up to 24 hrs later. This is reminiscent of the effect of swim stress on LTP-maintenance in the dentate gyrus, which causes changes in phosphorylation of [23] MAPK2, p38MAPK and pCaMKII within hours, but lasts for up to 24 hrs [44]. Of relevance is also the fact that some of the earlier studies involved recording from anaesthetized animals. It should be noted that anaesthesia itself is rather stressful [45], [46], so that the combination of stress exposure and anaesthesia most likely caused multiple surges of corticosterone. In view of the earlier reported metaplasticity of BLA neurons in response to consecutive pulses of corticosterone [47] - i.e. BLA neurons respond differently to the first pulse of corticosterone than to the second- interpretation of stress exposure in anaesthetized animals is complex.

While the present experiments showed that β-adrenergic and corticosteroid receptor activation at the time of stress changes network function such that induction of LTP is altered several hours later, understanding the mechanism by which these changes are accomplished is not trivial. Stress enhances the release of noradrenaline levels in the BLA [48], which through activation of β-adrenoceptors is known to enhance glutamatergic responses [49], [50], and presynaptic P/Q-type calcium currents [51], [52], probably increasing the likelihood to induce LTP postsynaptically (e.g. [53]). To what extent such effects are long-lasting, causing altered responses several hours after the brain has been exposed to enhanced levels of noradrenaline is unknown. The absence of changes in fEPSP amplitude suggests that increases in AMPA- or NMDA-receptor mediated responses most likely do not last for several hours. However, altered presynaptic calcium influx (albeit via L-type calcium channels) is known to activate a cAMP/PKA pathway which is necessary and sufficient for LTP induction in the cortico-LA pathway [54], [55]; this may be pertinent to the LA-BLA pathway as well. Activation of this downstream cascade could induce lasting effects on the ability to induce LTP, as well as the degree to which LTP is maintained [56], [57], [58], [59].

Restraint stress will also cause the release of corticosterone, as was confirmed in our study. Restraint stress and corticosterone lastingly enhance the frequency of mEPSCs in BLA neurons via a process requiring both MR and GR, but does not seem to affect the mEPSC amplitude [47], nor the amplitude of evoked EPSCs [50]. Moreover, corticosterone is known to enhance the amplitude of L-type calcium currents [60], shift the reversal potential of GABAa-receptor linked chloride channels towards more depolarized potentials [61] and enhance or at least not diminish, see [62]) firing frequency during depolarization. All of these effects have been observed several hours after a pulse of corticosterone and were found to be mediated by GRs. In particular the enhanced calcium influx, impaired firing frequency accommodation and reduced GABAa-receptor mediated inhibition, which develop several hours after the stress-induced rise in corticosterone level, may contribute to facilitated LTP at that time.

Interestingly, blockade of GRs or β-adrenoceptors at the time of stress reduced LTP induction beyond the level of control animals. This could point to interactions between the two transmitter systems, so that blockade of the one also affects the other, leading to cumulative effects. Besides noradrenaline, glucocorticoids may also interact with other neuromodulators such as corticotrophin releasing hormone and endocannabinoids to effectively alter amygdalar signaling after stress or glucocorticoid exposure [47], [63], [64], [65]. Other explanations may include compensatory, counter-regulatory processes (i.e. a shift in hormone balance or downstream signaling pathways) that consequently decrease plastic activity specifically under conditions of stress, although this remains speculative at this moment. Pharmacological blockade of the GR and/or β-adrenoceptor may reveal such potential compensatory mechanisms which under normal conditions may remain masked. These results are particularly interesting in a clinical setting for patients with aberrant GR and/or negative feedback function, such as may occur during major depression [66].

The effect of MR-blockade is very much in line with the presumed role of this receptor, i.e. maintaining the stability of synaptic transmission [5]. MRs, supposedly inserted into the plasma membrane, are indispensable to induce a rapid increase in mEPSC frequency, both in the CA1 hippocampal area and the BLA [47], [67]; these effects only develop with relatively high concentrations of corticosterone, such as may be reached after stress [67]. In the CA1 area, this rapid effect was associated with facilitation of LTP [68]. In the BLA, the MR-induced enhancement in mEPSC frequency is sustained [47]. This may explain why, several hours after stress, induction of LTP is facilitated, an effect that can be prevented by spironolactone applied prior to stress exposure. However, the fact that the MR-antagonist reduced the ability to induce LTP even in non-stressed animals suggests that actions via nuclear MRs also play a role in the likelihood to induce LTP in the LA-BLA pathway; these receptors are already extensively occupied under basal conditions, though a relatively small stress-induced recruitment on top of the tonically active receptor pool was described [9], [10].

At this time we can only speculate about the functional relevance of our observation that stress –via β-adrenoceptors and GR- promotes the ability to induce LTP in the LA to BLA projection some hours later, i.e. at a time that consolidation of the stressful event is thought to take place. If stress induced LTP-like processes in the LA-BLA pathway, later high-frequency stimulation of the same pathways would be expected to result in occlusion. This has indeed been described in the hippocampus [69]. However, it is very well possible that the actions of stress hormones are targeted towards other synaptic contacts in the BLA, and that noradrenaline and corticosterone extend the associative capacity within the BLA through facilitation of intersynaptic crosstalk, in a similar fashion as was recently proposed for β-adrenergic effects in CA1 neurons [70]. Probing LTP in the LA-BLA pathway is thus not only an (indirect) measure revealing lasting changes induced by stress in the BLA circuit at large, but also suggests that these changes might affect multiple inputs to this area.

The amygdala maintains a widespread network of direct and indirect projections to stress- and memory-related brain regions such as the hippocampus and prefrontal cortex [2], [71], [72]. Extensive evidence in humans and rodents suggests that via these projections the BLA plays a pivotal modulatory role in integrating the influences of stress on emotional memory formation [11], [35], [73]. For instance, recent literature indicates that stress or direct stimulation of the amygdala inhibits synaptic plasticity in the amygdala-prefrontal cortex pathway [25], [74], while hippocampus-dependent memory transmission in CA1 is impaired but enhanced in the dentate gyrus, the latter depending on stimulation strength and timing [27], [75], [76], [77], [78], [79], [80]. At the same time, BLA activation seems to prevent memory distortion during the consolidation of new, emotionally arousing information by inhibiting other memory-related processes through its connections with the hippocampus and prefrontal cortex [38]. In this way stress-induced changes in the function of the amygdala circuit may relay stress effects and modify neural signalling, plasticity and memory in connected brain regions. Collectively, this could underlie the observed behavioural effects of stress, involving emotional as well as cognitive (e.g. contextual) aspects of the event.
